# Some remarks on the argument appealing to nature against synthetic biology

**DOI:** 10.3389/fbioe.2024.1428832

**Published:** 2024-07-25

**Authors:** Ruipeng Lei, Yifan Peng, Yutian He, Jun Li

**Affiliations:** ^1^ School of Marxism, Center for Ethics and Governance of Science and Technology, University of Electronic Science and Technology of China, Chengdu, China; ^2^ School of Philosophy, Huazhong University of Science and Technology, Wuhan, China; ^3^ School of Economics, Huazhong University of Science and Technology, Wuhan, China; ^4^ Intellectual Property Development, Research Center China National Intellectual Property Administration, Beijing, China

**Keywords:** synthetic biology, nature, artifact, life, metaphysics, ethics

## Abstract

This paper will focus on analyzing the argument with appealing to nature against synthetic biology and provide a counter-argument against it through demonstrating the ambiguity of the concept of nature, denying the existence of a morally significant line between natural and non/unnatural, and disproving the allegations against synthetic biology raised by the argument appealing to nature. The paper consists of two parts following a brief introduction. The first part will describe the argument appealing to nature against synthetic biology, and identify the deficiencies of the argument *per se*, e.g., the ambiguity of the concept ‘nature’; and the problems in the morally significant line between the natural and the non/unnatural. The second part will discuss the allegations to synthetic biology stemming from this argument, e.g., committing metaphysical and ethical mistakes, and doing possible harms to the environment.

## 1 Introduction

To define synthetic biology as one of the emerging technologies is a difficult job. European Commission’s Scientific Committees (European Commission 2014) try to propose an operational definition based on the current knowledge about scientific, technical and commercial developments and a comprehensive survey of the existing scientific definitions of synthetic biology as follows:

“Synthetic biology is the application of science, technology and engineering to facilitate and accelerate the design, manufacture and/or modification of genetic materials in living organisms.” (European Commission 2014).

This definition include: 1) Modifying the natural or existing forms of life and transform them into new forms of life (currently on single cell microorganism), and 2) Create new forms of life directly from scratch which have never exist in nature and have new functions which any form of life in nature do not have by use of the knowledge of science, technology and engineering.

Different from traditional biology that studies the internal structure and functions of living organisms and from genetic engineering that recombines DNA through splicing technology, synthetic biology is committed to reassembling existing biological organisms or resembling artificial biological systems that do not exist in nature ‘from scratch’. As the pioneer, synthetic biologist Drew Endy states that in the new era of synthetic biology not only existing genes are described and analyzed but also new gene arrangements can be constructed and evaluated, and synthetic biology provides an opportunity to test the hypothesis that the genomes encoding natural biological systems can be ‘rewritten’, producing engineered surrogates that might usefully supplant some natural biological systems. (Endy, 2005). When Endy said ‘rewritten’ he was using the metaphor of a text. The transition from descriptive biology to genetic engineering and to synthetic biology can be paraphrased as the development from ‘reading’ to ‘editing’ to ‘writing’ DNA. (Boldt, 2013). If the task of traditional biology is to describe the phenomena of life, and the task of genetic engineering is to edit and modify life, then synthetic biology is devoted to ‘writing’ or ‘rewriting’ life software, designing and manufacturing new life forms, and realizing the transformation of biology from ‘reading’ to ‘writing’. If so, we can say that synthetic biology (incorporating ‘editing’) achieve a paradigmatic shift in biology.

Although genetically modified crops, animals (sentient animals in particular) and even human beings can be included into synthetic biology in theory, but there are quite different scientific-technological, philosophical and ethical issues which are interwoven with issues in agriculture, zootechnics, animal ethics, and human ethics. For the practical purpose, we will focus on synthetic microorganisms.

This paradigmatic shift is revolutionary. When synthetic biology enables human beings not only to understand and manipulate living things, but also to engineer and produce new creatures with new functions unprecedentedly in the history, it also brings great challenges to the traditional view of life, the human status in the nature, the relationship between humans and nature, the orders of thing in the cosmos, and causes great controversies about many issues including the legitimacy as well as the ethical justifiability and acceptability of synthetic biology. In this regard, some people with certain value system question the legitimacy of synthetic biology and construct ethical arguments against the research and application of synthetic biology.

## 2 The argument appealing to nature against synthetic biology and an analysis of its deficiencies

The argument against the legitimacy of synthetic biology, based on the appeal to nature, centers around the question of whether the design, production, and creation of living organisms fundamentally disrupt or undermine the relationship between humans and nature. This argument encompasses several points: firstly, it posits that organisms created through synthetic biology are unnatural and artificial (or man-made); secondly, it asserts that there exists a morally significant distinction between natural entities and artificial ones; thirdly, it argues that synthetic biology leads to metaphysical mistakes which place humanity in an inappropriate position within the universe; fourthly, it contends that synthetic biology results in ethical mistakes by devaluing life and overestimating human agency; finally, it suggests that synthetic biology causes harm or has the potential to cause harm to nature. Let us proceed with an analysis of the deficiencies of the argument appealing to nature as an argument in this part of the article, and then discuss the allegations to synthetic biology stemmed from the argument appealing nature in the next part.

## 3 Ambiguity of the concept nature

The argument that appeals to nature in order to challenge the legitimacy of synthetic biology can be formulated as follows:

1. The action that produces any living thing which does not occur naturally is morally wrong;
2. Engaging in synthetic biology produces living things that do not occur naturally; and
3. Therefore, engaging in synthetic biology is morally wrong.

We can observe that a key concept in this argument is “nature”. However, it is important to note that nature is a complex and highly ambiguous concept, making it impossible to form a valid ethical argument based on its multiple meanings. Synthetic biology aims to design and modify existing organisms or create new ones for specific tasks, either by using parts from existing organisms or by constructing organisms from non-living materials (“from scratch”). An article published in Nature, which does not oppose synthetic biology, defines it as having two subfields: “One use unnatural molecules to reproduce emerging behaviors from natural biology, with the goal of creating artificial life”, and “the other seeks interchangeable parts from natural biology to assemble into systems that act unnaturally” (Benner and Sismour, 2005). The authors all at once contrast the “naturalness” of original biology with the “unnaturalness” of synthetic biology. This has led some scholars to argue that synthetic biology represents a fundamental departure from natural biology rather than just an incremental difference. While traditional biological practices such as fermentation, animal husbandry, and agriculture utilize genetic material for human purposes, synthetic biology represents a new effort to synthesize living genetic materials for human use. It can then easily be seen as going beyond simply modifying or reorganizing natural givens to create living organisms and thus being accused of blurring the lines between organisms and artifacts, organic matter and synthetics, and living things and non-living entities (Lustig, 2013).

However, there is a significant challenge in appealing to the concepts of “nature” and “natural” to form an argument for a policy specific to synthetic biology that differs from traditional biotechnologies. This is due to the fact that synthetic biology is dedicated to creating “non/unnatural” organisms, which raises questions about whether research and application of synthetic biology should be approved.

The term “nature” originates from the Latin word natura, derived from the verb meaning “to be born”. According to classical accounts, the English word “nature” has had three senses over time. In the 13th century, nature referred to the essential quality or character of something, such as the nature of a person. From the 14th century onwards, it was also used to denote the inherent force directing both the world and human beings, as in “the way of nature”. It was not until relatively recently—specifically in 17th century English usage—that the word “nature” came to refer to the physical world as a whole. As such, it encompasses various meanings in reference to both humans and biophysical reality.

The concept of “nature” is polysemantic and elusive. Numerous natural and social scientists are vying to contribute to the formulation of the concept of “nature” as they see fit. How can we reconcile the polysemantic senses of “nature” in order to construct a compelling argument against synthetic biology? For instance, American environmentalist James Proctor identified five views on nature: nature as evolution, nature as emergence, nature as malleable, nature as culture, and nature as sacred (Proctor, 2009). Proctor notes that the first two views prevalent in the physical, biological, and behavioral sciences; the latter two arise in the social sciences, humanities, and theology; while malleable nature straddles both sciences and humanities (Proctor, 2009). In reality, there are many more views of nature than those enumerated by Proctor. The diverse viewpoints on appealing to “nature” or “the natural” encompass a complex blend of facts and beliefs, descriptions and regulations, emotions and intuitions. How can experts from various disciplines come together to establish a shared concept of “nature” and its moral significance for constructing a valid ethical argument that distinguishes synthetic biology from traditional biology? This seems unattainable.

Ethical arguments that serve as the foundation for decision-making must withstand rigorous philosophical analysis. However, appealing to nature and the natural presents challenges in terms of precise analysis. This is due to the fact that an individual’s conception of nature often encompasses a multitude of complex issues that are difficult to isolate and analyze individually, particularly when they operate primarily on an intuitive and emotional level. Furthermore, assessing the persuasiveness of appeals to nature requires consideration of historical and cross-traditional interpretations. Specific views on nature and the natural are frequently contentious within traditional frameworks and across different cultural perspectives.

According to the recent findings by de Graeff et al. (2022), the concept of nature (or natural) and unnaturalness (the unnatural) is ubiquitous to be discussed on and referred to in food products materials, in beauty product advertisements, at philosophy conferences, in newspaper articles or in scholarly publications. Nonetheless, it is not always clear what is meant by ‘nature’ and ‘(un)naturalness’. Even in the scholarly or even philosophical publications the terms or concepts of nature and unnaturalness are shown to be elusive, imprecise, value-laden, culture specific, and changed over time. They are used in diverse ways, to refer to different things, and there are widely varying opinions on what is natural and what is not. It is why they are referred to “essentially contested concepts”.

The authors (de Graeff et al., 2022) further point out that there are major overarching concepts in the arguments regarding nature: nature as 1) the non-human; 2) entangled nature; and 3) nature as the essential characteristics of a thing. For the first overarching concept, nature is defined as that which “would exist without humans and would exist in the way it does without humans” (Birnbacher, 2019) The ‘natural’ conceptualized this way thus refers to the living and non-living entities that are independent from human beings or human influence. It is often contrasted to the ‘unnatural’, the ‘artificial’ or the ‘artifactual” (de Graeff et al., 2022). In de Graeff and her colleagues’ view, this ‘all-or-nothing’ conception of nature as the non-human entails that all these national parks in many countries and some other wilderness areas would not be considered nature, because humans play an essential role in shaping wilderness areas worldwide. If one adopts this conception of nature as the non-human, then it would be impossible to delineate the natural from the unnatural, leaving no true sense of untouched nature remaining in the world (de Graeff et al., 2022).

The second overarching concept of nature pertains to what can be termed as ‘entangled nature’. According to this concept, humans are not separate from nature but rather an integral part of it. In other words, humans and nature are intertwined in various ways. The entire physical world is governed by the laws of physics, chemistry, and evolutionary biology. This is the nature we refer to when we mention ‘natural laws’ or ‘Mother Nature’ (de Graeff et al., 2022). This idea has been associated with religion as much as with modern science. In a religious context, the observation of order and principles in nature has led to the belief that it must have been designed by a supernatural entity, i.e., God. In a scientific context, it is linked to what has been referred to as the ‘world machine’; the perspective of nature as a grand machine driven by specific principles (Clarke, 1993).

The third overarching concept of nature is that of nature as the essential characteristics of a thing, i.e., “that which makes it what it is and not something else, its ontological identity card” (Daston, 2019). This concept of nature relates to the scientific study of taxonomy, which categorizes different organisms into different organic species that have characteristic features, properties and tendencies (Daston, 2019). Different accounts of this concept exist that each provide their own specific view on what they take nature to consist of. Some conceptions ground this concept of nature in a common biological essence, whereas others ground it in the way a particular type of being behaves.

Based on the accounts made by de Graeff and her colleagues as well as other scholars it can be expected that the elusive, imprecise, value-laden, culture specific and changed over time concept of nature may not withstand the rigorous test of philosophical analysis. Therefore, it may not serve as a reliable ethical argument for developing proper policies in synthetic biology.

The philosophical challenge known as the “Is/Ought issue” undermines attempts to utilize appeals to nature in making moral judgments about synthetic biology. Such judgments cannot be derived solely from descriptions of nature. In everyday life, describing and evaluating activities are not easily separable. Since ancient times, from the Greek philosophers Stoics (as they claim that “Living according nature”) onwards, nature has been invoked as the original source of universal order, providing justification for moral choices and actions. (Durand et al., 2023). However, developments in philosophy and science have called into question the conflation of experience with morality, as evidenced by Hume’s critique of arguments that unjustifiably move from description to prescription.

For instance, in nature, there exists both the law of the jungle and reciprocal symbiosis. The question then arises: which one should serve as our moral compass? Therefore, when making moral judgments, we must refer to a normative standard that transcends nature; otherwise, we would fall into the “naturalist fallacy.” While drawing on nature has deep historical roots and merits consideration of its potential moral implications, the intricacies of the concept of nature and the tension between many different ways of thinking about nature have raised doubts about the validity of arguments based on nature in policy discussions regarding synthetic biology. It is challenging to see how appealing to nature can function persuasively as a basis for policies that outright prohibit or severely restrict synthetic biology. The assessment of a specific research program or project’s policy implications must be conducted on a case-by-case basis to make informed decisions considering factors such as risks and benefits, individual and societal wellbeing, respect for stakeholders, environmental impact, equitable access, *etc.*, without relying on an ambiguous, vague, complex and contentious concept like nature to determine whether synthetic biology or other emerging sciences/technologies should be promoted, permitted or prohibited. (Lustig, 2013).

In his influential book Toward More Natural Science: Biology and Human Affairs (Kass, 1985) American philosopher Kass proposed the idea that modern science could become a “more natural science”. For him nature is given, and she is a purposive, well-ordered whole aligning with Aristotelian and quasi-Kantian view of nature. Kass insisted that‘Natural’ and ‘more natural’ mean here only ‘true (or “truer”) to life’ as found and lived,” (Kass, 1985, xi, 346–348). He asked the question“Is our growing dominion over living nature compatible with respecting our own given nature?“, however, he did not further ask the question: “Do we have a ‘given’ nature?” Kass believed a more natural science could move “from nature to ethics”. “A more natural science might be useful for ethics,” because it would show how ethics is “part of nature,” and so “the natural, rightly understood, might even provide some guidance for how we are to live” (xi, 346-48).

In our view, the fundamental question at hand is whether a connection can be established between nature and morality. Nature does indeed speak to us; but does she provide legible clues to our moral duty? The answer is definitely “no”! Nature speaks with many, often contradictory voices: the behavior of the black widow spider toward her mate (she eats him) is no less natural than the gentler lessons that Kass adduces (Kimball 1985). His claim of “from nature to ethics” is also inconsistent with his legitimate disavowal of the notion that “precise rules of conduct [might be] deducible from even the fullest knowledge of nature—no sensible person holds that such rules can be simply ‘read off’ from the natural record.” (Kimball 1985). In 2002, with the publication of his essay “The permanent limitations of biology” (Kass, 2002, 296–297) Kass drepudiated his “more natural science” of biological ethics. He spoke of “the insufficiency of nature for ethics” and “the difficulty in looking to biology--even a more natural science more true to life--for very much help in answering the questions about how we are to live”. Although Kass’ argument regarding the connection between nature and ethics lacks validity, his work on a more natural science still holds value as “an invitation to reflection,” as he himself stated.

## 4 Problems in the morally significant line between the natural and the non/unnatural

Advocates of the argument based on nature assert that there exists a morally significant boundary between natural entities and non/unnatural (or artificial) ones (or artifacts). Natural entities are those that occur in nature and are not created by humans. In contrast, unnatural entities are man-made and cannot be found in nature. Synthetic entities, whether living or non-living, are typically produced in laboratories through the combination of various chemicals or prepared compounds and substances, or through DNA segments or modified genomes. Non-natural entities are not naturally occurring on Earth and are instead created by humans. In this context, non-natural can be considered synonymous with man-made or with unnatural or artificial in the sense of “not existing in nature.” However, it does not align with the other sense of unnatural or artificial, which refers to something that is not in accordance with nature or the normal course of natural events; in other words, abnormal or perverse. For example, one might say “The altered landscape looks strange and unnatural” or refer to an “artificial smile.” Some proponents argue that there are two categories of things in nature: natural and non/unnatural or artificial/artefactual (man-made). They assert that there is a morally significant distinction between the two: Natural is good while non/unnatural or man-made is not inherently good. The following is a construction of the appeal to nature argument: (Curtis, 2020):

That which is natural is good or right.

N is natural.

Therefore, N is good or right.

That which is unnatural is bad or wrong.

U is unnatural.

Therefore, U is bad or wrong.

According to the formula above synthetic organisms are deemed “unnatural” and therefore not good or even bad, then should be rejected. There exists a normative consensus in environmental philosophy that places higher value on natural entities compared to man-made ones, although this consensus may be subject to debate.

Long before the emergence of synthetic biology, numerous environmental philosophers had already issued warnings about the potential demise of nature (McKibben 1989: 48). They argued that novel technologies were nature-replacing and posing a threat to the very existence of the natural world (Lee, 2003), and suggested preparing for a post-nature era (Vogel, 2002). For these thinkers, “nature” represents a separate and untamed world that exists independently from humans, who are subject to its rules of birth and death. Many positions in environmental philosophy are based on the fundamental proposition that there is a morally significant line between natural entities on one hand, and unnatural or artificial ones on the other. The writings of influential environmental philosophers such as Leopold (1949), Elliot (1982), Rolston (1986), Rolston (1988), Katz (1992), reflect a widespread intuition that unmodified nature holds moral significance. Central to environmental philosophy is the effort to define what is natural and distinguish it from what is non-natural or artificial. In this endeavor, they often turn to Aristotle’s characterization of a natural object in The Physics as one which possesses an inherent principle of movement and stationariness (192b8-11) (Hardie and Gaye, 1941). In contrast, an artifact lacks its own source of production; this principle resides externally in something else—typically human intentionality (192b28). The external source to which Aristotle refers is the intentional action of a human. Artificial things thus display the presence of human intention. Natural things do not. These thinkers all emphasize that wild nature’s naturalness is independent from human intention in an Aristotelian sense, asserting that this independence carries moral weight.

Those scholars who oppose biotechnology and synthetic biology heavily rely on Aristotelian distinctions and the moral overlays described above. For instance, they argue that the true cost of biotechnology, including synthetic biology, lies in their ability to systematically transform naturally occurring biotic beings into artificial ones. As technologies that replace nature, they claim that molecular biotechnology constitutes a radical threat to the ontological category of the natural, wherein natural living beings with higher value are replaced by artificial living beings with lower value (Lee, 1999: 114). However, it is important to note that humans have been creating biotic artifacts for thousands of years. The hybridization of crops and the domestication of animals, as well as using artificial selection to supplement natural selection, have been widely accepted in various cultures without posing a challenge to agriculture.

There are two key points that need to be addressed. The first point is the difficulty in defining what is natural and unnatural, and drawing a clear distinction between the two. Different individuals may have varying interpretations of naturalness. Furthermore, the concept of what is considered natural or unnatural evolves over time.

One way to define natural things is as those found in nature. However, if we consider the natural world to encompass the whole of the natural or physical world, i.e., all physical objects and phenomena, this definition fails to effectively delineate between what we perceive as natural and unnatural. For instance, synthetic polymers, particle accelerators, and robots are just as much a part of the physical world as wild panda or forests. This broad interpretation includes many items that are typically deemed unnatural.

Conversely, if our perception of nature is limited to traditional countryside elements, then the definition becomes too narrow and excludes numerous entities. Another perspective defines natural processes as those occurring without human intervention. This explanation accounts for the naturalness of phenomena such as photosynthesis, pollination, animal reproduction, aging, and death. However, this viewpoint categorizes many human activities requiring human intervention as “unnatural”. Yet cooking and writing poetry are not unnatural, nor are natural human reproduction and natural pregnancy meeting this criterion. Therefore, it proves challenging to distinctly classify items into either being purely natural or entirely unnatural.

The apparent simplicity of Aristotelian distinction is ultimately revealed to be an illusion, as the inherent difficulties in distinguishing the natural from the non-natural or artificial become evident. In his 1874 essay on Nature, John Stuart Mill (Mill, 1874) highlighted a troubling paradox: while all human actions are considered natural due to their origin, none of these actions transcend natural laws. However, Mill also recognized that everything humans do leaves nature in a non-natural state. Therefore, it becomes clear that the Aristotelian distinction is inadequate for capturing such nuances and cannot serve as a normative imperative for environmental philosophers seeking to preserve a pure or non-humanized nature (Nuffield, 2015; Lei et al., 2018).

The second point is that it is not necessarily appropriate to associate natural things with positive labels and unnatural things with negative labels. Some argue that using this distinction to differentiate between ethically acceptable and unacceptable technologies is highly questionable.

As previously mentioned, scholars who oppose synthetic biology often rely on Aristotle’s distinction between the natural and the artificial. However, we do not believe that Aristotle would support their argument in this context. The distinction made by Aristotle pertains to ontology rather than morality. Precisely, it is these scholars who imposed the moral sense on the distinction between the natural and the artificial.

In discussions surrounding science, technology, and medicine, the assertions about the ethical implications of naturalness are often deemed arbitrary or unreasonable. Bioethicists sometimes immediately dismiss appeals to nature. For instance, when debating the opposition to assisted reproductive technologies, bioethicists John Harris and Soren Holm rejected the appeal to naturalness (Harris and Holm, 2000). They argued that many unnatural interventions are integral to modern medicine and widely regarded as good and valuable aspects of human activity. Doctors and medical scientists could do nothing if they accepted arguments based on what is considered natural or unnatural and all moral implications that these arguments entail, such as we should not intervene in natural things, thereby the whole practice of medicine is deemed as unnatural (Savulescu and Webber, 2014).

The philosopher Frances Kamm (Kamm, 2005) argues that many aspects of nature are inherently negative. Cancer cells, HIV, tornadoes, and toxins all exist within the realm of nature. The question then arises: are they inherently good or sacred? It is important to recognize that nature and goodness are two distinct categories; natural things may not necessarily be good, while things deemed as good may not always be considered natural, instead, they may be deemed as unnatural. Bioethicist Guido de Wert (de Wert, 2000) has opposed the use of a distinction between natural and unnatural in guiding ethical decision-making within novel sciences, technologies, and medical practices. He has pointed out that some individuals condemn reproductive technologies as morally wrong due to their being “unnatural”. However, he contended that the argument asserting “X is wrong because it is unnatural” lacks justification as it fails to clearly differentiate between natural and unnatural actions while also failing to demonstrate that unnatural actions are morally wrong.

## 5 Allegations to synthetic biology stemming from argument appealing to nature

### 5.1 On the allegation of committing metaphysical mistakes by synthetic biology

Opponents of synthetic biology argue that it subverts the relationship between human and nature by making erroneous metaphysical claims, thus committing a metaphysical mistake (Kaebnik, 2013:53–57). It is contended that whether it be nature itself or the divine creator (God) who has carefully orchestrated the universe, there are certain realms into which humans should not intrude. For instance, scholars at the Stanford Center for Biomedical Ethics assert (Cho et al., 1999) that synthetic biology is criticized for undermining the concept of life as unique by defining life solely in terms of DNA and reducing it to mere biological characteristics. Additionally, synthetic biology has been accused of employing reductionism to strip away the particularity of life (Cho et al., 1999). German scholars Joachim Boldt and Oliver Müller (Boldt and Müller, 2008) argue that synthetic biology has transformed humans from mere “describers” and “manipulators” of natural life to “creators”. They assert that the transition from manipulation of what already exists to creation of what does not exist is a decisive shift, signifying a fundamental change in the way we treat nature. Synthetic biology seems to advocate for a new relationship between humans and the nature: one where humans can control nature and adapt it to human needs, rather than humans have to adapt themselves to nature This shift will inevitably lead to a change in the way humans treat nature—no longer revering it with respect or fearing its power, but instead using it as a blank piece of paper, and nature becomes the object of human domination. (Boldt and Müller, 2008).

We will argue that it is unfounded to criticize synthetic biology for committing a metaphysical mistake. As previously mentioned, Joachim Boldt and Oliver Müller contend that synthetic biology has shifted humans from being mere “describers” and “manipulators” of natural life to becoming “creators,” signifying a fundamental shift in the way we treat nature (Boldt and Müller, 2008). Critics of synthetic biology argue that the universe have an inherent order, whether it is established by nature itself or by a divine creator at the time of creation. This established relationship between humans and nature cannot be subverted. Furthermore, despite Darwin’s theory of evolution having long negated this natural or divinely-arranged order, these critics still firmly believe that there exists a threshold for the relationship between humans and nature, beyond which this relationship will be subverted. For instance, they contend that conventional biotechnology has not surpassed this threshold, whereas synthetic biology has done so. Now the question is: On what basis can we assert that synthetic biology in its design, production, and manipulation of living organisms exceeds this threshold and fundamentally alters the relationship between humans and nature?

Let us now consider another reproach posed by those scholars who oppose synthetic biology: its metaphysical understanding of life is deemed to be wrong. Specifically, they argue that synthetic biology undermines specialness or particularity of life by demonstrating that life is purely a material phenomenon, and that a living organism is simply a complex combination of physical and chemical components. In the first scholarly article on the ethical issues of synthetic biology, for instance, Mildred Cho and her coauthors contend that by defining life in terms of DNA, synthetic biology reduces life to a single biological feature and therefore “may threaten the view that life is special” (Cho et al., 1999). When Craig Venter’s team synthesized the genome of M. mycoides, inserted it into an M. capricolum cell, and successfully produced a line of reproducing M. mycoides cells, this achievement was heralded as falsifying the notion that living things are “endowed with some sort of special power, force or property” (Caplan, 2010).

However, Kaebnik (2013) argues that the mere act of creating a living organism in a laboratory does not necessarily strip it of any special properties, as these can be conferred upon it in other ways. If there is a God capable of bestowing the particularity of life to the creatures in the swamp, then he can also give this particularity to the creatures produced in the laboratory. In a sense, scientists have been generating living organisms for quite some time; whether through *in vitro* fertilization where life emerges from the combination of gametes in a test tube to form an embryo, or through successful animal mating resulting in new life. While synthetic biology alters the method by which life is created, it does not change the fundamental fact that new life is being brought forth. Therefore, any special properties typically associated with naturally occurring microorganisms can also be found within synthetic counterparts.

The central concern at hand revolves around two distinct issues for decision-makers: One issue is whether life possesses specialness or particularity separate from non-living entities, and the role that humans play in the universe. This is an ontological or metaphysical issue, which holds less relevance to our actions pertaining to synthetic biology. The other is the ethical issue of whether we should support or dismiss synthetic biology, which is directly and urgently relevant to our action on the field. These are two distinct matters. Even if synthetic biology has transformed humans from mere manipulators of life to creators of life, as previously described, it also signifies a shift in the role of humans within the universe. As for whether the change of this metaphysical status of humans indicates that metaphysical mistake has been committed to by synthetic biologists, opinions vary. However, labeling humans as life creators (or “playing God”) merely suggests that they are surpassing their established role in the universe. In order to address the issue of the role that humans should play in the universe and whether synthetic biology involves a metaphysical mistake, it is necessary to conduct a philosophical analysis of the concept of “human role in the universe”, establish criteria for evaluating whether the actions taken by synthetic biologists deviate from this criterion, and gather evidence to prove any such deviation. Given the divergence of worldviews and belief systems among philosophers, it is challenging for them to reach a consensus in the near future. However, we can anticipate that the ethical issue of whether to support or dismiss synthetic biology can be effectively addressed by trans-disciplinary scholars, including philosophers. Through public reason, these scholars can work towards reaching a consensus on this matter. (Mandle, 2013). We are not able to discuss public reason in detail here.

### 5.2 On the allegation of committing ethical mistakes by synthetic biology

Synthetic biology are also blamed for making ethical mistakes: Synthetic biology produces a view of the relationship between human and nature that conflicts with so-called basic concepts of moral practice. (Kaebnik, 2013:57–60). Boldt & Müller argue that synthetic biology’s description of organisms as machine-like artifacts challenges the link between “life” and “value,” and may ultimately lead to a weakening of society’s respect for higher life forms. (Boldt and Müller, 2008). They argue that synthetic biology has changed people’s conceptions of life and human’s role in the universe, which is unethical.

The criticism that synthetic biology has made ethical mistakes is also unfounded. As mentioned above, Boldt and Müller (2009) argue that synthetic biology transforms humans from “describers” and “manipulators” of natural life to “creators”, and thus conflicts with underlying concepts of moral practice, according to which the description of organisms as machine-like artifacts challenges the link between “life” and “value”, devalues life, and ultimately leads to a weakening of society’s respect for persons with higher form of life. Furthermore synthetic biology may change the concept of human agency, in which humans are no longer just the manipulator of nature, but the creator or modifier of nature, leading to hubris.

As for synthetic life devalues life, we can begin by pointing out that there is no reason to assume that scientific research exploring life will compel us to devalue life. The fact that an organism is created in the laboratory does not diminish its value as less than that of a naturally occurring organism. As bioethicist Arthur Caplan put it, after the announcement of the synthesis of mycobacteria, the value of its life is not impaired and devalued by the possibility of understanding its function. (Caplan, 2010). Those who hold a reductionist view on bacterial life do not necessarily lead to the debasement of higher forms of life. When we create synthetic life, it exhibits characteristics and abilities that are unique to living organisms, rather than just chemical changes, which possibly leading people to devalue life. A survey on public attitudes towards synthetic biology indicates that people are generally unconcerned about the creation and modification of single-celled organisms, despite the fact that synthetic biology has the potential to create and modify more complex forms of life (Royal Academy of Engineering, 2009). However, attitudes towards single-celled life differ significantly from those towards higher forms of life such as humans. The historical perspective of viewing animals as machines does not raise any moral concerns leading to a devaluation of human life (Kaebnik, 2013).

To the extent that life has value distinct from inanimate objects, further philosophical analysis of the statement “life has value” is necessary. Unlike inanimate objects, all forms of life possess the property or ability of self-organization, metabolism, survival, reproduction, and evolution. However, these properties or abilities alone are insufficient to confer intrinsic/inherent (used interchangeably) value upon life itself. In this context, ‘value’ should denote intrinsic value rather than mere external instrumental value. The term ‘value’ is best understood in terms of ‘moral status,’ which allows us to recognize the complexity inherent in the general statement that ‘life has value.’ When we assert that humans possess the highest moral status (and thus the highest value) among all life forms, it is because humans have their own unique capacity for self-consciousness, reason and emotion, social relations, and so on Kelly and Morar (2014)).

When we assert that sentient animals possess varying degrees of moral status (varying degrees of value), this determination is based on their capacity to experience pain and pleasure, as well as some possessing a lower degree of self-consciousness, reason, emotion, social relations, etc. compared to humans. However, the characteristics or abilities related to self-organization, metabolism, survival, reproduction, and evolution alone cannot determine the inherent value or moral status of life unless one allows for the possibility of value or moral status to be negative. For instance, can we attribute value or moral status to HIV, SARS virus, avian influenza virus, coronavirus and *Aedes aegypti* which causes pandemics? Apparently not. There is clearly no moral problem with the eradication of viruses or the *A. aegypti* mosquito. However, those who oppose synthetic biology on the grounds that all forms of life hold value are conflating two distinct categories of issues. The question of whether all forms of life possess unique characteristics such as self-organization, metabolism, survival, reproduction, and evolution - which differentiate them from non-living entities - is an ontological or metaphysical issue. On the other hand, the question of whether all forms of life (including HIV, SARS virus, avian influenza virus, coronavirus, and *A. aegypti*) hold inherent value is a moral issue.

To the extent that synthetic biology may lead humans to become hubristic, i.e., overestimate our ability to understand and manipulate the world, this does not appear to be an ethical issue in itself, or at most a matter of virtue (humility being a virtue). A person who is overly confident, arrogant, and hubristic in their estimation of their abilities does not necessarily act against ethical norms. However, it can be argued that hubristic individuals may use their abilities recklessly, potentially leading to unintended negative consequences. If we are concerned about the potential undesirable outcomes of hubris, then this becomes a moral question related to the consequences of actions rather than a specific argument about how synthetic biology might alter our relationship with nature. If synthetic biologists were arrogant enough to create not only unicellular organisms but also animals capable of feeling pain and pleasure or even fully conscious human beings through synthetic action, such actions would likely face public condemnation and regulatory prohibition. The ethical issue at that time revolves around the question of whether we should synthesize sentient animals or fully conscious human beings, rather than being simply a matter of hubris. If scientists are content to conduct synthetic biology research with unicellular organisms in a lab, their hubris does not necessarily raise ethical concerns. However, it is important to note that science has a tendency to challenge ideas about life and our role in the universe. For instance, Copernicus removed human beings from the center of the universe, Darwin blurred the sharp division between humans and animals, and German chemist Friedrich Wöhler’s synthesis of urea challenged the concept of “vital force”. Did they act wrongly? No! They did not degrade morality; on the contrary, they took humanity a great step forward in morality. (Kaebnik, 2013).

### 5.3 On the allegation of possible harms to the environment

Scholars who raise objections to synthetic biology are concerned about its potential harmful impact on the environment, particularly in relation to the changing dynamic between humans and nature. The crux of these concerns lies in the belief that the environment should be safeguarded not only for its inherent value to humanity but also as a responsibility entrusted to humans. Consequently, it is imperative for human beings to adopt an attitude of reverence and gratitude towards natural entities.

Environmental philosopher Christopher Preston has expressed opposition to synthetic biology along the same line. He argues that synthetic biology blurs the distinction between natural organisms and artifacts, a differentiation which traditional molecular biotechnology does not compromise. The key disparity lies in the fact that traditional biotechnology operates by modifying existing organisms’ genomes through gene deletion or addition, whereas synthetic biology aims at “creating an entirely new organism,” thereby transgressing a fundamental boundary cherished by environmentalists: deviating from Darwinian evolution’s core principle of progression through modification. This departure may potentially yield adverse effects on nature (Preston, 2008). Scholars who object to synthetic biology are concerned about its harmful impact on the environment associated with changing the relationship between humans and nature. The cornerstone of such concerns is the belief that the environment should be protected, not only to ensure its benefits for humans but also to be protected by humans. For this reason, human beings should have an attitude of reverence and gratitude for natural things.

It is a moral imperative for synthetic biologists and regulators to carefully monitor the potential negative impacts of their work upon the environment, and make a great efforts to prevent and reduce these impacts, however, so far the claim that synthetic biology has a detrimental impact on nature lacks empirical evidence. In contrast to Preston’s assertion, there are numerous successful instances of synthetic biology that do not demonstrate any serious or irreversible damage to the environment. For instance, the synthesis of artemisinin has led to tens of millions of malaria patients worldwide being cured without any evidence indicating harmful effects on the environment. On the contrary, it has had a positive impact by helping local areas where artemisinin plants are cultivated to move away from monocropping and restore crop diversity (Ro et al., 2006).

The issue of the human-nature relationship, which necessitates the formulation of appropriate public policies, often entails quantifiable harm to the natural world, particularly severe and irreversible damage. For instance, when the last passenger pigeon is killed, the species becomes extinct. However, creating organisms does not necessarily cause environmental harm. Specifically, engineered organisms are confined to laboratories, factories, and farms without impacting the surrounding nature. In fact, synthetic biology can be advantageous for environmental improvement. For example, by synthesizing the genome of the passenger pigeon and placing it in a surrogate egg, we could potentially revive this species. However, the revival of mammoth by synthesizing the genome is another matter. We have to assess the impacts of synthesizing life on the environment case by case. Furthermore, synthetic biology has the potential to reduce pollution and mitigate climate change by enabling bacteria to produce clean energy and substances that eliminate environmental pollutants or absorb carbon dioxide.

However, critics of synthetic biology argue that while the technology itself may not directly harm the environment, it promotes a mindset that disrupts the balance between humanity and nature. This perspective suggests that nature should be manipulated to meet human needs, rather than humans adapting to coexist with nature. However, this objection to synthetic biology is unfounded as this tendency can be attributed to all technologies. Both adapting nature to accommodate human needs and adjusting human behavior to align with natural processes are essential. For example, in China, we have successfully redirected water from the south to benefit millions of people in the north. Simultaneously, it is crucial for us to prioritize water conservation efforts. These two actions are both opposite and complementary to each other. In Chinese idiom, it is referred to as “xiang fan xiang cheng” (相反相成). This argument against synthetic biology cannot hold up in itself unless there are objective evidences to prove it makes the environment worse. The issue of petroleum fuel production and consumption is so immense and severe that even if new methods of fuel production through synthetic biology are discovered, there still needs to be a reduction in fuel consumption. In summary, given the magnitude of the environmental crisis, even if we research and implement synthetic biology to create new clean energy sources, there still needs to be a shift in human behavior to align with natural reality. We do not have a binary choice between adapting nature to human behavior and adapting human behavior to nature.

The research and application of synthetic biology may pose potential risks to the environment, which is not fundamentally different from the development of other technologies. For instance, the utilization of synthetic algae for fuel production has the potential to deplete nutrient sources in ecosystems, diminish flora and fauna diversity, displace or negatively impact native algal or microbial populations, disrupt aquatic ecosystems and their food web dynamics. Additionally, the transfer of synthetic or modified genetic material into other organisms could result in a strain of algal microorganisms causing disease in non-human organisms, stimulating the growth of environmentally harmful algae, and generating unknown or novel transgenic-related toxins (Hewett et al., 2016).

These potential environmental impacts must be carefully assessed, and measures must be taken to avoid and minimize them. This process is no different from assessing the environmental impact of other technologies when conducting research and applying them. Therefore, these potential environmental impacts should not serve as a reason to oppose synthetic biology; rather, they should prompt us to improve our methods for conducting environmental risk assessment and management in the development of synthetic biology.

We must emphasize that the potential environmental impacts of synthetic biology, as discussed above, are not fundamentally different from those of other technologies. However, this does not imply that these impacts should be underestimated. While synthetic biology has the potential to contribute to basic research in biology and life sciences, as well as address global social issues such as food security, nutrition, oil production, medicine/vaccine development, and environmental protection, it also poses significant risks to human health and the environment. These risks must be anticipated in order to develop effective prevention and management measures. Of particular concern are biosafety and biosecurity risks. Biosafety risks pertain to the potential harmful effects on workers and the environment resulting from accidental interactions with hazardous biological agents. Biosecurity risks involve the potential misuse of synthetic biology for purposes such as bioterrorism, biowarfare or bioattacks through genome manipulation in micro-organisms. (Gómez-Tatay and Hernández-Andreu, 2019).

## 6 Conclusion

Based on the accounts provided above, it can be concluded that the argument against synthetic biology based on appeals to nature is invalid. The concept of nature is polysemantic, elusive, and ambiguous. There is no clear demarcation line between what is considered natural and non/unnatural, and the distinction is not morally significant. Distinguishing between naturalness and unnaturalness pertains to ontology or metaphysics rather than morality or ethics. The allegations against synthetic biology posed by the argument appealing to nature are without a sound moral basis. Discussions about the value of natural living beings *versus* the disvalue of synthetic life forms do not contribute to formulating sound policies regarding synthetic biology.

Synthetic biology could have profound implications, and it is crucial to focus on relatively simple organisms to ensure that the biological changes it causes are also relatively straightforward. These simpler organisms are not sentient, much less conscious, and do not require informed consent for research. Therefore, there are no too much regulatory barriers to synthetic biology research, allowing for an accelerated pace of advancement in the field. The creation of entirely new forms of life, organisms that do not naturally exist, represents a “second genesis.” We agree with philosopher Edward Regis when he asserts that one of the main points of synthetic biology is philosophical in the original sense - the love of knowledge including physics and metaphysics as well as philosophy in the present sense. The most profound and thought-provoking question raised by the construction of an artificial living cell is an ancient riddle: What is life? (Regis, 2008).

## Data Availability

The original contributions presented in the study are included in the article/Supplementary Material, further inquiries can be directed to the corresponding author.
